# Evaluation of the mycobacteriophage amplification assay for detection of physically and physiologically stressed mycobacterial cells *in vitro*

**DOI:** 10.1186/1471-2334-14-S3-O19

**Published:** 2014-05-27

**Authors:** Eddy SG Cheah, Yee Thin Tan, Yii Han Tay, Seow Hoon Saw

**Affiliations:** 1 Department of Biological Science, Faculty of Science, Universiti Tunku Abdul Rahman, Kampar, Perak, Malaysia; 2 Department of Biomedical Science, Faculty of Science, Universiti Tunku Abdul Rahman, Kampar, Perak, Malaysia

## Background

Currently used laboratory detection methods for *Mycobacterium tuberculosis* are either slow or low in sensitivity, and therefore there is a need for a more reliable and rapid method. This study was done to evaluate the efficiency of the mycobacteriophage amplification assay in detecting mycobacterial cells that were exposed to the stresses (desiccation, UV radiation, and nutrient starvation) that simulate those in tuberculous droplet nuclei.

## Methods

Exponential-phase *Mycobacterium smegmatis* (non-pathogenic surrogate model for *M. tuberculosis*) cells were exposed to a particular stressor and then subjected to the phage and colony-forming unit (CFU) assays; the numbers of plaque-forming unit (PFU) and CFU yielded were then compared to those for the unexposed control. We also investigated the effects of treating stressed cells with culture supernatants (containing resuscitation-promoting factors) on their phage infectivity and culturability on solid medium.

## Results

Only desiccation and UV exposure decreased PFU significantly (~5x10^6^ to 5x10^5^ PFU/mL and 7.7x10^6^ to 3.4x10^6^ PFU/mL, respectively), whereas all three stressors affected culturability by approximately ten folds (10^6^-10^8^ to 10^5^-10^7^ CFU/mL). These suggest that most stressors affect the culturability of mycobacterial cells more than their phage infectivity. With prior culture supernatant treatment, only desiccated cells showed improved PFU and CFU yields (1.5x10^6^ to 3.1x10^6^ PFU/mL and 4.4x10^6^ to 5.8x10^6^ CFU/mL, respectively).

## Conclusion

Hence, there is preliminary evidence that the phage assay might be superior to culture for detection of stressed mycobacterial cells that are in the non-culturable but viable state, and prior resuscitation of stressed cells could potentially improve the assay sensitivity.

